# The Paradoxical Effect of Repeated Body Checking on Subjective Uncertainty

**DOI:** 10.1002/eat.24315

**Published:** 2024-10-29

**Authors:** Mor Ben Zaken Linn, Eyal Kalanthroff, Noam Weinbach

**Affiliations:** ^1^ School of Psychological Sciences, University of Haifa Haifa Israel; ^2^ Department of Psychology The Hebrew University of Jerusalem Jerusalem Israel

**Keywords:** body checking, eating disorders, OCD, repeated checking, uncertainty

## Abstract

**Objective:**

Body checking is considered a behavioral expression of the core psychopathology of eating disorders (EDs), namely, overvaluation of body weight and shape. Compulsive checking is motivated by a desire to increase a sense of certainty regarding feared outcomes. Paradoxically, studies showed that repeated checking acts to reduce certainty, forming a vicious cycle. No previous study examined whether the same principle applies for body checking. This study filled this gap by examining the causal effect of repeated body checking on memory certainty regarding checked body parts.

**Method:**

In a laboratory‐based study, 77 female participants without an ED checked the size and shape of six body parts. Their objective memory regarding which body part was last checked, and subjective certainty about this memory were assessed. Then, one group of participants continued to engage in repeated body checking, and another group repeatedly checked a neutral object. Finally, all participants completed the six body parts checking procedure again, and their objective memory and memory certainty were re‐assessed.

**Results:**

In both checking groups, objective memory regarding the last body part checked was unaffected by the type of checking performed. Importantly, certainty about memory dropped considerably only among those in the repeated body‐checking group.

**Discussion:**

The findings provide the first empirical evidence of a paradoxical effect demonstrating that repeated body checking reduces certainty about checked body parts. The study implies that repeated body checking reduces the quality of information obtained through checking and, as such, could potentially motivate further checking.

**Summary**

- Compulsive body checking is a common symptom of eating disorders that negatively affects psychological well‐being.
- Body checking often occurs to increase certainty about feared outcomes.
- This study revealed a paradoxical effect in which repeated body checking reduces, rather than increases, certainty about checked body parts.
- The results imply a potentially vicious cycle through which repeated body checking and the feeling of uncertainty maintain one another.

## 1 Introduction

Repeated body checking is a behavior aiming at gaining information about one's body shape, weight, or size and is considered a transdiagnostic symptom of eating disorders (EDs; American Psychiatric Association 2013). Body checking may include examining one's body in the mirror while focusing on disliked body parts, frequently weighing, and scrutinizing different body parts by touching and pinching (Shafran et al. 2004). Compulsive body checking is associated with various EDs symptoms.

Multiple studies report that a tendency to engage in body checking is associated with negative affect, disordered eating, and body dissatisfaction (see meta‐analysis in Walker, White, and Srinivasan 2018). Increasing body checking in healthy individuals causes higher body dissatisfaction (Shafran et al. 2007; Walker et al. 2021), fear of uncontrollable weight gain (Bailey and Waller 2017), and attentional bias to body‐related cues (Smeets et al. 2011). Body checking was suggested as a safety behavior that downregulates negative emotions in the short term but maintains body‐related anxiety over time (Haase, Mountford, and Waller 2011). Cognitive‐behavioral models of EDs consider body checking as a behavioral expression of the “core psychopathology” of EDs, namely, overvaluation of body weight and shape (Fairburn, Cooper, and Shafran 2003). Given the pivotal role played by body checking in EDs symptomatology, it is important to understand the mechanisms perpetuating it.

A possible venue to investigate how body checking is maintained is by harnessing knowledge from disorders in which repeated checking is a central symptom, such as obsessive‐compulsive disorder (OCD; Rachman 2002). In OCD, it was theorized that checking aims to reduce uncertainty regarding feared outcomes (Rachman 2002; Tallis 1997). However, repeated checking paradoxically increases uncertainty, which encourages further checking, forming a vicious cycle. The paradoxical effect of repeated checking on uncertainty was replicated in multiple experimental studies (see meta‐analysis in Van Den Hout et al. 2019). These studies asked healthy individuals to engage in repeated checking of external stimuli (e.g., check if virtual gas rings are open or closed). When participants were asked about the last items checked, their memory was unaffected. However, their subjective certainty about their memory decreased dramatically compared to participants who did not repeatedly check those items.

Repeated body checking may follow a similar principle. Specifically, body checking occurs to reduce the feeling of uncertainty regarding feared outcomes like weight gain (Kesby et al. 2017), but repeated body checking may increase, rather than reduce, uncertainty. However, no previous study examined the causal influence of body checking on uncertainty. A recent correlational study showed that higher self‐report levels of intolerance of uncertainty, a common trait in EDs (Brown et al. 2017), are associated with a higher tendency to engage in body checking among individuals recovered from anorexia nervosa and healthy controls (Bijsterbosch et al. 2022). Thus, there are indications that body checking and uncertainty are related.

In a laboratory‐based study, we examined the influence of repeated body checking on objective memory and subjective certainty regarding checked body parts. In line with an experimental psychopathology approach, the study included women without EDs because a stronger causal inference regarding mechanisms can be obtained by mimicking abnormal processes in non–afflicted individuals (Jansen 2016; Van Den Hout, Engelhard, and McNally 2017). Specifically, if repeated body checking is a mechanism that increases body‐related uncertainty, independently of other EDs characteristics or symptoms, then anyone who engages in repeated body checking should show elevated body‐related uncertainty, regardless of the presence or absence of an ED.

Participants were assigned to one of two checking groups. One group engaged in repeated body checking, and another repeatedly checked a neutral object. Before and after the checking process, participants in both groups checked six body parts and were asked to recall the last body part checked (objective memory) and rate how certain they were that they checked this body part (subjective memory certainty). We hypothesized that objective memory regarding the last body part checked would be unaffected in both groups. However, subjective certainty was expected to reduce in the repeated body‐checking group.

## 2 Method

### 2.1 Participants

The study included 77 female participants aged 18–39 randomized to a body‐checking (*N* = 39) or an object‐checking group (*N* = 38) using “Research Randomizer” (www.randomizer.org). Inclusion criteria were fluent Hebrew speakers with an age range of 18–45 years old. Exclusion criteria were a current or previous history of an ED (indicated via self‐report) and a body mass index (BMI; kg/m^2^) lower than 18.5 or higher than 30. A power analysis using G*Power (Faul et al. 2007) indicated that a sample of 62 participants would be sufficient to detect within‐between factors interaction with power > 80%, an alpha of 0.05, and an effect size estimate of *η* ^2^_p_ = 0.12. The effect size was determined by an unpublished study in our lab.

### 2.2 Self‐Report Measures

To ensure no baseline group differences in measures that could potentially impact the results, participants completed questionnaires assessing ED symptoms (Eating Disorder Examination‐Questionnaire; EDE‐Q; Fairburn and Beglin 1994), the tendency to engage in body checking (Body‐Checking Questionnaire; BCQ; Reas et al. 2002), and intolerance of uncertainty (Intolerance of Uncertainty Scale, short form; IUS‐12; Carleton, Norton, and Asmundson 2007). Psychometrics of these questionnaires are presented in Supporting Information A.

### 2.3 Procedure

The study was approved by the School of Psychological Sciences Ethics Committee at the University of Haifa (approval number 476/22). Ads informed potential participants that the study involved weighting and body checking while wearing a sleeveless shirt. Participants signed informed consent and completed self‐report questionnaires online. An in‐person lab session was scheduled at least 24 h afterward.

In the laboratory, participants were asked to wear a sleeveless shirt, which was provided if they did not bring one. Participants were informed that the experimental room was not recorded and that they would be alone during the experiment to ensure their privacy. Participants followed automated audio recordings delivered via speakers. At the beginning of the experimental session, participants completed a six‐body parts checking procedure (detailed below) to assess the dependent measures. Then, they completed the experimental manipulation (detailed below), after which they completed the six‐body parts checking procedure again. Lastly, participants' height and weight were measured, and they were debriefed.

#### 2.3.1 Six‐Body Parts Checking

Before and after the manipulation, participants in both groups were asked to stand in front of a full‐sized body mirror and inspect their whole bodies visually. Through audio recording, participants completed instructed checking of six body parts: face, arms, wrists, stomach, waist, and thighs, each for a fixed duration of 9 s (see detailed instructions in Supporting Information B). The order of the checked body parts was randomized across participants. Subsequently, participants were asked to sit in front of the computer and mark the last body part checked from a checkbox of the six checked body parts (i.e., objective memory measurement). Then, participants rated *how certain they were that it was the last body part checked* (i.e., subjective certainty measurement) on a visual analog scale (VAS) from 0 (not certain at all) to 100 (very certain).

#### 2.3.2 Repeated Checking Manipulation

After the first six‐body parts checking procedure, participants began the repeated checking manipulation. In the body‐checking group, participants were instructed to stand in front of the mirror and examine and measure the six body parts previously examined by following audio instructions. In the object‐checking group, participants were asked to examine and measure six parts of a toy truck (door, steps to the driver seat, wheels, pole, box, roof) by following audio instructions. Both experimental groups performed 48 instructed checking trials with eight repetitions of each body/object part. The repeated checking manipulation lasted 7.5 min in both groups.

### 2.4 Data Analysis

Baseline differences between the groups in age, BMI, and all self‐report measures were assessed using independent *t* tests. Given the binary score (correct/incorrect) of the objective memory question, Wilcoxon Signed‐Ranks tests were conducted separately for each group to assess pre‐ to postmanipulation changes in the percentage of participants who correctly recalled the last body part checked. Subjective certainty data were analyzed using a mixed‐model analysis of variance (ANOVA) with mean subjective certainty VAS score as the dependent measure, time (pre–manipulation/post–manipulation) as the within‐subject variable, and group (body‐checking/object‐checking) as the between‐subject variable. Partial eta square was used as an effect size estimate, with 0.02, 0.06, and 0.14 representing small, medium, and large effect sizes, respectively, according to conventions (Cohen 1988).

## 3 Results

All data have been made publicly available at the Open Science Framework repository (OSF): https://osf.io/x4mv5/?view_only=476038c212344183bd7b23533d203efe. As presented in Table 1, the groups did not differ in age, BMI, and all baseline self‐report measures.

**TABLE 1:** Age, BMI, and self‐report questionnaires: Means and statistical values.

	Body checking (*n* = 39)	Object checking (*n* = 38)	*t*(75)	*p* value	Cohen's *d*
Age (years)	23.89 (3.93)	23.5 (3.24)	0.48	0.63	0.11
BMI	22.98 (3.62)	24.54 (3.38)	1.96	0.05	0.45
EDE‐Q	2.51 (1.25)	2.96 (1.40)	1.49	0.14	0.34
BCQ	38.56 (10.04)	42.47 (14.48)	1.52	0.13	0.35
IUS‐12	32.1 (8.35)	33.82 (10.66)	0.79	0.43	0.18

*Note*: Standard deviations appear in parentheses.

Abbreviations: BMI, body mass index; EDE‐Q, eating disorder examination‐questionnaire; BCQ, body checking questionnaire; IUS‐12, intolerance of uncertainty scale, short form.

### 3.1 Objective Memory

**FIGURE 1:**
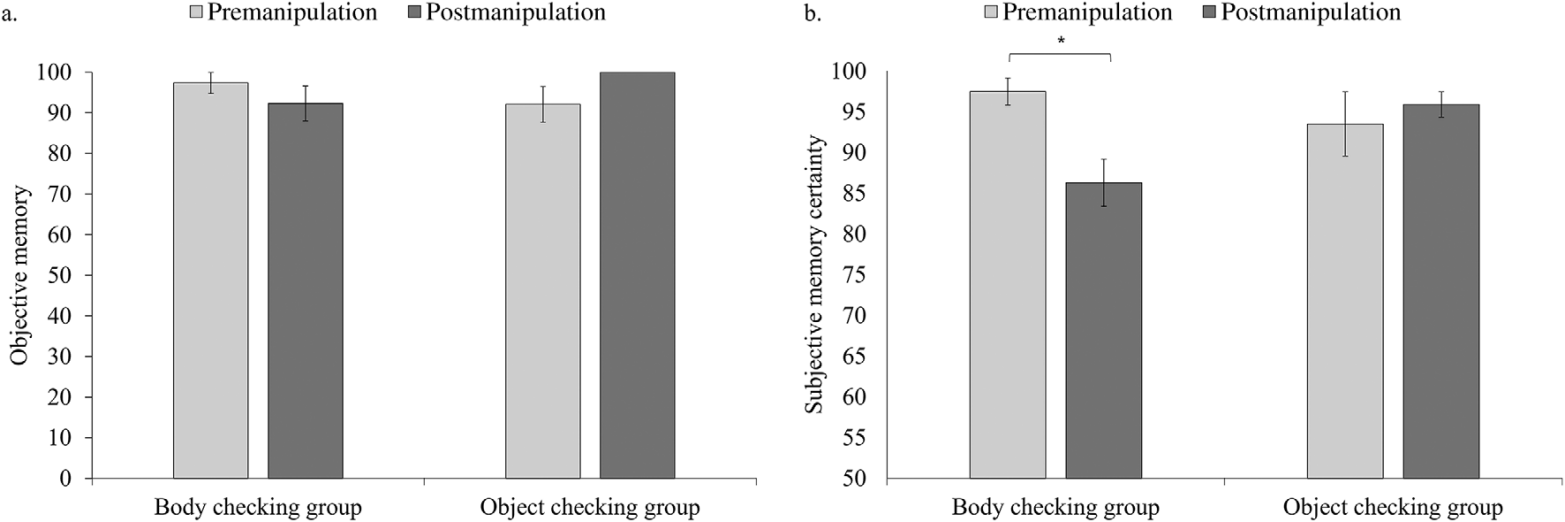
The y‐axis in (a) represents the percentage of participants who correctly recalled the last body part checked in the 6‐item body‐checking procedure. The y‐axis in (b) represents participant's memory certainty ratings regarding the last body part checked. The x‐axis represents the experimental group. Light gray bars represent the pre–manipulation measurement (i.e., before repeated checking), and dark gray bars represent the post–manipulation measurement (i.e., after repeated checking). Error bars represent standard errors. *p < 0.01.

The general percentage of participants who correctly recalled the last body part checked was 95.45%. As hypothesized, the analyses revealed no significant change in the percentage of correct recall from pre‐to‐post manipulation in both the body‐checking group, *Z* = 1.0, *p* = 0.32, Cohen's *d* = 0.029, and the object‐checking group, *Z* = 1.73, *p* = 0.08, Cohen's *d* = 0.51 (see Figure 1a).

### 3.2 Subjective Certainty

The ANOVA revealed a main effect for time, *F*(1, 75) = 4.0, *p* = *0*.049, *η* ^2^_p_ = 0.05, and no main effect for Group, *F*(1, 75) = 0.81, *p* = *0*.37, *η* ^2^_p_ = 0.01. Importantly, the analysis revealed a significant Time × Group interaction, *F*(1, 75) = 9.52, *p* = *0*.003, *η* ^2^_p_ = 0.11. As hypothesized, there was a significant decrease in certainty ratings from pre‐to‐post manipulation only in the body‐checking group, *F*(1, 75) = 8.13, *p* = 0.007, *η* ^2^_p_ = 0.09, and not in the object‐checking group, *F*(1, 75) = 1.56, *p* = 0.22, *η* ^2^_p_ = 0.02 (see Figure 1b). Between‐group contrasts showed no group difference in memory certainty at pre–manipulation, *F*(1, 75) = 1.44, *p* = 0.23, *η* ^2^_p_ = 0.02. However, at post–manipulation, memory certainty was significantly lower in the body‐checking group compared to the object‐checking group, *F*(1, 75) = 5.03, *p* = 0.03, *η* ^2^_p_ = 0.06. None of the self‐report measures moderated the effect of Group on changes in certainty (all ps > 0.21). A tendency to engage in body checking was positively correlated with EDs symptoms (*r* = 0.69, *p* < 0.001) and intolerance of uncertainty (*r* = 0.37, *p* < 0.001; see analyses in Supporting Information C and D).

## 4 Discussion

The goal of this study was to examine the impact of repeated body checking on subjective certainty. As expected, objective memory regarding checked body parts was unaffected in both checking groups. However, subjective certainty regarding the memory of checked body parts significantly dropped among participants who engaged in repeated body checking, with a medium‐to‐large effect size. Conversely, in the active control group that repeatedly checked a neutral object, memory certainty regarding the last body part checked remained high.

OCD research suggests that repeated checking of external stimuli (e.g., gas stoves, light bulbs, locks) reduces memory certainty regarding checked items (Van Den Hout et al. 2019; van Uijen and Toffolo 2015). This paradoxical effect, where more checking leads to greater uncertainty and thus more checking, is thought to perpetuate compulsive checking. Our study is the first to demonstrate that the same principle applies when the stimulus being checked is one's own body, suggesting that repeated body checking is likely not distinct from other forms of compulsive checking behaviors. Specifically, despite no objective reason for memory distrust given the simplicity of recalling the last body part checked, repeated checking of body size and shape caused participants to feel less certain about their memory. This suggests that repeated body checking diminishes the quality of information obtained and, consequently, reduces certainty about that information. If checking diminishes the quality of information obtained, this may increase the urge to check even more. Indeed, body checking in EDs was suggested to follow an urge to gain certainty regarding feared outcomes such as weight gain (Kesby et al. 2017). Additional research is required to determine if body‐related uncertainty increases the urge to engage in body‐checking and actual‐checking behaviors.

Cognitive‐behavioral therapy for EDs suggests ways to reduce body checking in treatment (Fairburn, Cooper, and Shafran 2003). However, these do not address the potential links between body checking and uncertainty. A potential implication of this study is to encourage therapists to discuss the paradoxical nature of repeated body checking with clients, emphasizing that it is not an effective way to obtain information about the body. While the initial intent may be to gain certainty, repeated checking of body size and shape actually leads to greater uncertainty. Understanding this paradox could help increase motivation to reduce body‐checking behaviors in treatment.

This study was conducted on a nonclinical sample, presenting both a strength and a limitation. It is a strength because if repeated body checking inherently increases body‐related uncertainty, independently of other EDs characteristics, then this effect should occur in anyone who engages in repeated body checking. As such, mimicking typical EDs behaviors in non–afflicted individuals offers more precise insights into causal mechanisms (Jansen 2016). However, it is also a limitation because the reported effects may differ in patients with EDs. Nonetheless, ethically, it would be inappropriate to experimentally increase body checking in ED patients. Additional limitations include the inability to determine the long‐term effect of repeated body checking on memory certainty, the inability to generalize the findings to individuals who do not identify as women, children, adolescents, or adults over 40, and the reliance on self‐reported ED diagnoses as an exclusion criterion. Lastly, the study was not preregistered.

To conclude, the study presents novel findings on the effect of repeated body checking on subjective certainty. These findings provide an important step toward a better understanding of the mechanism through which body checking is perpetuated. Future research is encouraged to assess the theoretical model according to which body checking and intolerance of uncertainty exacerbate one another in a way that maintains EDs symptomatology.

## Author Contributions

**Mor Ben Zaken Linn:** conceptualization, data curation, formal analysis, investigation, writing – original draft. **Eyal Kalanthroff:** conceptualization, writing – review and editing. **Noam Weinbach:** conceptualization, funding acquisition, supervision, writing – review and editing.

## Conflicts of Interest

The authors declare no conflicts of interest.

## Supporting information


**Data S1.** Supporting Information.

## Data Availability

All data have been publicly available at the Open Science Framework repository (OSF) and can be accessed at https://osf.io/x4mv5/?view_only=476038c212344183bd7b23533d203efe.
