# Supplementary material for: The Paradoxical Effect of Repeated Body Checking on Subjective Uncertainty
Source: Int J Eat Disord. 2024 Oct 29;58(1):238–42. doi: 10.1002/eat.24315 (PMC11784847; doi:10.1002/eat.24315)
Supplement: Supplementary file 1 — Data S1. Supporting Information. [file EAT-58-238-s001.docx]

# Supplementary Materials

1. **Self-report measures.**

Eating Disorder Examination- Questionnaire (EDE-Q; Fairburn & Beglin, 1994). The EDE-Q includes 36 items that assess eating disorder symptoms. The EDE-Q provides a global score and four subscales (restraint eating, eating concerns, weight concerns, and shape concerns). The questionnaire has demonstrated high validity in assessing the core attitudinal features of ED psychopathology in the general population (Fairburn & Beglin, 1994). We used a Hebrew version of the EDE-Q that was validated in a previous study (Zohar et al., 2017). Cronbach’s α in the present study was .88.

Body-Checking Questionnaire (BCQ; *Reas et al., 2002)*. This self-report measure consists of 23 items assessing body-checking behaviors. The BCQ has good test-retest reliability and internal consistency (Reas et al., 2002). Two bilingual translators translated the questionnaire into Hebrew through forward translation and independent back-translation. Cronbach’s α in the present study was .90.

*Intolerance of Uncertainty Scale, short form (IUS-12;**Carleton et al., 2007)*. The IUS-12 is a 12-item short-form of the original 27-item Intolerance of Uncertainty Scale (Freeston et al., 1994) that measures reactions to uncertainty, ambiguous situations, and the future. The IUS-12 has demonstrated excellent internal consistency and convergent validity with the original 27-item scale (Carleton et al., 2007). The Hebrew version of the questionnaire has shown good discriminant and predictive validity (Cohen et al., 2022). Cronbach’s α in the present study was .92.

## Experimental instructions verbatim.

The checking instructions were delivered through pre-recorded audio instructions. The order of the body parts checked was randomly presented among participants.

Pre-manipulation six-body parts checking:

- Face checking: “Pull your face back, pressing your chin toward your neck, and look at your face in the mirror”.
- Arm checking: “Pinch and grasp the skin under one of your arms”.
- Wrist checking: “Encircle your left wrist using the fingers of your right hand”
- Stomach checking: “Pinch and touch the skin on your stomach”.
- Waist checking: “Pinch and touch the skin on your right waist”.
- Thigh checking: “Encircle one of your thighs using both of your hands”.

Post-manipulation six-body parts checking:

- Face checking: “Pinch the skin on your cheeks”.
- Arm checking: “Encircle one of your arms using the fingers of your other hand”.
- Wrist checking: “Encircle your right wrist using the fingers of your left hand”.
- Stomach checking: “Pull your stomach in as much as possible, and look at it in the mirror”.
- Waist checking: “Pinch and touch the skin on your left waist”.
- Thigh checking: “Pinch the skin on the back of your thighs”.

Manipulation

*Body checking group instructions:*

The order of the body parts checked was randomly presented among participants. Each checking trial was repeated four times.

- Face checking:
  - “Pull your face back, pressing your chin toward your neck, and look at your face in the mirror”.
  - “Pinch the skin on your cheeks”.
- Arm checking:
  - “Pinch and grasp the skin under one of your arms”.
  - “Encircle one of your arms using the fingers of your other hand”.
- Wrist checking:
  - “Encircle your left wrist using the fingers of your right hand”.
  - “Encircle your right wrist using the fingers of your left hand”.
- Stomach checking:
  - “Pinch and touch the skin on your stomach”.
  - “Pull your stomach in as much as possible, and look at it in the mirror”.
- Waist checking:
  - “Pinch and touch the skin on your right waist”.
  - “Pinch and touch the skin on your left waist”.
- Thigh checking:
  - “Encircle one of your thighs using both of your hands”.
  - “Pinch the skin on the back of your thighs”.

*Object checking (toy truck) group instructions:*

The order of the toy truck parts checked was randomly presented among participants. Each checking trial was repeated 4 times.

- Door checking:
  - “Open the truck’s door and measure its height using your right hand”.
  - “Open the truck’s door and measure its height using your left hand”.
- Step checking:
  - “Run your finger along the top step of the truck and feel it”.
  - “Run your finger along the bottom step of the truck and feel it”.
- Wheels checking:
  - “Encircle one of the rear wheels with your right hand”.
  - “Encircle one of the rear wheels with your left hand”.
- Pole checking:
  - “Lift the truck's box and measure the pole underneath it using your right hand”.
  - “Lift the truck's box and measure the pole underneath it using your left hand”.
- Box checking:
  - “Measure the height of the truck's box using your right hand”.
  - “Measure the height of the truck's box using your left hand”.
- Roof checking:
  - “Measure the length of the truck's roof using your right hand”.
  - “Measure the length of the truck's roof using your left hand”.

## C. Moderation analysis

Multiple regression analysis was conducted to examine whether self-report measures of EDs symptoms (EDE-Q), intolerance of uncertainty (IUS-12), and body checking (BCQ) moderated the effect of Group on pre-to-post manipulation changes in certainty. The analysis included interaction terms between Group and each of the self-report measures to test for moderation effects. As presented in Table 2, none of the self-report measures significantly interacted with Group in predicting changes in certainty (all ps > .21).

**Table 2.** Statistical values of the interactions between Group and self-report measures on pre-to-post manipulation changes in certainty.

| Interaction factor | B | *β* | *t(69)* | *p*-value |
| --- | --- | --- | --- | --- |
| Group * EDE-Q | 1.52 (4.71) | .13 | 0.32 | .75 |
| Group * IUS-12 | -0.23 (0.52) | -.21 | -0.45 | .66 |
| Group * BCQ | -0.66 (0.53) | -.77 | -1.26 | .21 |

*Note*. Standard errors appear in parentheses. The table presents unstandardized beta coefficients (B) and standardized beta coefficients (β). EDE-Q: Eating Disorder Examination- Questionnaire; IUS-12: Intolerance of Uncertainty Scale, short form; BCQ: Body-Checking Questionnaire.

## D. Correlational analysis

A correlation analysis examined relationships between EDs symptoms, the tendency to engage in body-checking, and intolerance of uncertainty. The analysis included results from both groups and revealed (see Table 3) that scores on the BCQ (body-checking tendencies) were positively associated with EDE-Q scores (eating disorders symptoms) and IUS-12 scores (intolerance of uncertainty). Furthermore, EDE-Q scores were positively associated with IUS-12 scores.

**Table 3.** Correlations between self-report measures.

|  |  | EDE-Q | IUS-12 | BCQ |
| --- | --- | --- | --- | --- |
| EDE-Q | Pearson's r | 1 |  |  |
|  | p-value | — |  |  |
| IUS-12 | Pearson's r | .34 | 1 |  |
|  | p-value | .002 | — |  |
| BCQ | Pearson's r | .69 | .37 | 1 |
|  | p-value | < .001 | < .001 | — |

*Note*. The analysis included all participants (n = 77). EDE-Q: Eating Disorder Examination- Questionnaire; BCQ: Body-Checking Questionnaire; IUS-12: Intolerance of Uncertainty Scale, short form.
